# SARS-CoV-2 and gastrointestinal diseases

**DOI:** 10.3389/fmicb.2023.1177741

**Published:** 2023-05-30

**Authors:** Ailong Sha, Yi Liu, Xuewen Zhao

**Affiliations:** ^1^School of Teacher Education, Chongqing Three Gorges University, Chongqing, China; ^2^School of Biology and Food Engineering, Chongqing Three Gorges University, Chongqing, China

**Keywords:** SARS-CoV-2, gastrointestinal diseases, mechanism, treatment, gastrointestinal

## Abstract

**Background:**

Severe acute respiratory syndrome coronavirus-2 (SARS-CoV-2) is the causative agent of the novel coronavirus disease (COVID-19) pandemic, which has caused serious challenges for public health systems worldwide.

**Literature review:**

SARS-CoV-2 invades not only the respiratory system, but also the digestive system, causing a variety of gastrointestinal diseases.

**Significance:**

Understanding the gastrointestinal diseases caused by SARS-CoV-2, and the damage mechanisms of SARS-CoV-2 to the gastrointestinal tracts and gastrointestinal glands are crucial to treating the gastrointestinal diseases caused by SARS-CoV-2.

**Conclusion:**

This review summarizes the gastrointestinal diseases caused by SARS-CoV-2, including gastrointestinal inflammatory disorders, gastrointestinal ulcer diseases, gastrointestinal bleeding, and gastrointestinal thrombotic diseases, etc. Furthermore, the mechanisms of gastrointestinal injury induced by SARS-COV-2 were analyzed and summarized, and the suggestions for drug prevention and treatment were put forward for the reference of clinical workers.

## 1. Introduction

Novel coronavirus disease 2019 (COVID-19), an acute respiratory infectious disease caused by severe acute respiratory syndrome coronavirus-2 (SARS-CoV-2), has become a global and high incidence respiratory disease, with the most common clinical manifestation of pulmonary infection. However, some studies have shown that the stool samples of COVID-19 patients showed persistent positivity for SARS-CoV-2, and the duration was longer than that of respiratory samples, suggesting that SARS-CoV-2 may spread the infection between the gastrointestinal tract (van Doorn et al., 2020). Some patients had gastrointestinal symptoms, such as nausea, vomiting, and diarrhea (Zeng et al., 2022), but none of them had the imaging features of COVID-19. In severe cases, diseases, such as gastric ulcer, colitis, intestinal obstruction, and intestinal ischemia, were also present. In addition, with the emergence of different variants of SARS-CoV-2, the gastrointestinal symptoms of COVID-19 patients become more and more common, and some gastrointestinal symptoms are combined with or prior to the onset of respiratory symptoms, and COVID-19 patients with gastrointestinal symptoms are more likely to develop acute respiratory distress syndrome and liver damage, and have a poor prognosis (Ye Q. et al., 2020; Chen et al., 2022). If the gastrointestinal diseases caused by SARS-CoV-2 are not properly recognized, and the best treatment opportunity is missed, the patient’s risk of death may increase. Therefore, it is of great significance to understand the gastrointestinal diseases caused by SARS-CoV-2 and the damage mechanisms of SARS-CoV-2 to the gastrointestinal tract and gastrointestinal glands for treating gastrointestinal infections caused by SARS-CoV-2. This review mainly summarizes a variety of gastrointestinal diseases caused by SARS-CoV-2, and discusses possible prevention and treatment programs based on the SARS-CoV-2 pathogenesis. It is hoped that this review can provide a reference for clinical workers to diagnose the patients with SARS-CoV-2-related gastrointestinal diseases during the COVID-19 pandemic, and also provide ideas for the prevention and treatment of gastrointestinal disorders in patients diagnosed and cured of COVID-19, so as to further reduce the risk of SARS-CoV-2 infection and the mortality rate of COVID-19 patients.

## 2. Gastrointestinal diseases caused by SARS-CoV-2 infection

After SARS-CoV-2 infection, in addition to a series of respiratory diseases, gastrointestinal symptoms are becoming more and more common, and are related to the severity of the patient’s condition. Some studies have calculated the probability of various gastrointestinal symptoms during the COVID-19 pandemic, and found that the detection rate of some gastrointestinal diseases was increased. The following is a brief description of the gastrointestinal diseases caused by SARS-CoV-2 (Table 1).

**Table 1:** Gastrointestinal diseases caused by SARS-CoV-2 infection.

Disease type	Proportion	Categorical statistics/Counts
Gastrointestinal inflammatory lesions	41.30%	38/92
Ulcerative lesions of the gastrointestinal tract	14.13%	13/92
Gastrointestinal bleeding	7.61%	7/92
Gastrointestinal thrombotic lesions	36.96%	34/92

The presidential count in the table refers to the total number of patients with gastrointestinal diseases counted in this paper, that is, the sum of the corresponding gastrointestinal diseases found in the reference literature. The proportion shown is calculated based on the proportion of a particular disease to the total number.

Table 1 is cited from Alberca et al. (2021) and Marasco et al. (2021).

### 2.1. Gastrointestinal inflammatory lesions

The binding of spike protein (S Pro) to angiotensin-converting enzyme 2 (ACE2) is the first step for SARS-CoV-2 to enter cells. ACE2 is not only widely distributed in alveolar cells, but also gastrointestinal epithelial cells. ACE2 has strong vasoconstrictive and pro-inflammatory effects, which can cause various gastrointestinal inflammatory diseases in COVID-19 patients, such as erosive gastritis and hemorrhagic gastritis, leading to gastrointestinal bleeding. Gastrointestinal symptoms in patients with COVID-19 mainly include diarrhea, abdominal pain, nausea, and vomiting; A study by Zeng et al. (2022) showed that the severe disease rate of patients with gastrointestinal symptoms of COVID-19 was more than 40%. Abdominal pain was associated with a 2.8-fold increased risk of severe COVID-19 infection and may be used as clinical predictor of severe COVID-19; the relationship between diarrhea and the severity of COVID-19 was regionally different; nausea and vomiting were limited in their association with severe COVID-19. Massironi et al. (2020) collected statistics from 38 COVID-19 patients undergoing gastrointestinal endoscopy. Among the 38 patients, 37 required hospitalization. Eight patients were admitted to an intensive care unit. Most of the patients had lesions, including duodenal ulcer in 5 cases and erosive gastritis in 4 cases. The main findings during colonoscopy included segmental colitis associated with diverticulosis in 5 cases, histologically confirmed colon ischemia in 4, diffuse hemorrhagic colitis in 1. Another follow-up study showed that 11 COVID-19 patients with gastrointestinal symptoms all had an initial symptom of gastritis, including intestinal mucosal inflammation in 8 cases, intestinal ulcers or erosion in 2 cases, and colonic mucositis inflammation in 2 cases, and some patients still presented with the gastrointestinal symptoms and intestinal damage 6 months after discharge (Xie et al., 2022). In addition, multiple COVID-19 patients have been diagnosed with colitis, including segmental colitis, hemorrhagic ulcerative colitis, and ischemic colitis (Massironi et al., 2020). Vanella et al. (2021) performed endoscopy on 106 patients with COVID-19 (33% admitted to the intensive care unit; 44.4% reported gastrointestinal symptoms). The most prevalent upper gastrointestinal abnormalities were ulcers (25.3%), erosive/ulcerative gastroduodenopathy (16.1%), and petechial/hemorrhagic gastropathy (9.2%). In the lower gastrointestinal endoscopy, 33.3% showed ischemic colitis. The first report on hemorrhagic colitis caused by SARS-CoV-2 gastrointestinal infection originated from a 71-year-old woman who developed hemorrhagic colitis without any respiratory symptoms. She was diagnosed with SARS-CoV-2 infection after detection (Carvalho et al., 2020), which was thought to be a gastrointestinal complication caused by SARS-CoV-2 infection. In addition, Imperatore et al. (2021) reported a case of COVID-19 patient without a history of gastrointestinal disease before illness, who presented with bloody diarrhea at a visit 1 month after illness and was subsequently diagnosed with ulcerative colitis (UC). Another study has found that a COVID-19 patient showed UC symptoms in the colon and ileum after SARS-CoV-2 turned negative, which can literally reflect the reason why some infected patients still showed positive stool of SARS-CoV-2 after turning negative (Calabrese et al., 2020). It has been reported that the leading cause of UC is related to the changes in intestinal microbes (Taxonera et al., 2021). Besides, pancreatitis is also one of the gastrointestinal diseases caused by SARS-CoV-2, but its overall prevalence is lower compared with other gastrointestinal case reports. The diagnosis of acute pancreatitis requires at least two of the following three signs: abdominal pain; amylase or lipase >3 times the upper limit of normal; characteristic findings of imaging diagnosis. A study has shown that 9 of 52 COVID-19 patients (17%) have been detected with mild pancreatic injury, whose symptoms were mainly characterized by an increased incidence of pancreatitis and diarrhea, as well as increased pancreatic serum enzymes (Wang F. et al., 2020). Liu F. et al. (2020) studied the pancreatic injury of 121 COVID-19 patients. About 1 to 2% of non-severe COVID-19 patients and 17% of severe COVID-19 patients had pancreatic injury. ACE2 was expressed in the pancreas of normal people. In addition, the expression level of SARS-CoV-2 in pancreas was slightly higher than that in lung, indicating that SARS-CoV-2 may combine with ACE2 in pancreas to cause pancreatic injury. Bacaksız et al. (2021) assessed the elevation of amylase and lipase in 1378 COVID-19 infected patients and its relationship to COVID-19 severity. Of the 1,378 patients, 316 had some degree of amylase elevation (23%), and pancreatitis was detected in only 6 patients (1.89%). In the remaining patients, elevated amylase and lipase were found to be associated with the severity of COVID-19 infection. It has been reported that in 8.5–17.3% of COVID-19 patients, elevated levels of amylase and lipase, among others, indicated pancreatic injury and were higher in patients with severe COVID-19 (Bruno et al., 2021). Therefore, more attention should be paid to the pancreas in SARS-CoV-2 infected patients, especially in severe cases. In summary, the gastrointestinal inflammation caused by SARS-CoV-2 infection can be manifested as inflammation of a single organ or combined with inflammation in different durations, and some gastrointestinal symptoms may precede other clinical symptoms. Therefore, in the clinical treatment of COVID-19 patients, attention should be paid to the occurrence of gastrointestinal inflammation, and regular check-ups should be carried out during the recovery period after discharge.

### 2.2. Ulcerative lesions of the gastrointestinal tract

In addition to causing the gastrointestinal inflammation, ACE2 can also have a synergistic effect with SARS-CoV-2, causing necrosis and degeneration of the gastrointestinal mucosa, leading to ulcerative lesions. Therefore, the gastrointestinal ulcerative lesions also occur frequently in COVID-19 patients, and can be diagnosed by endoscopic evaluation. Several studies have reported that it can appear in various parts of the tongue, hard/soft palate, lips and buccal mucosa, oropharynx, esophagus, stomach, duodenum, and large intestine. In the autopsy of a COVID-19 patient, two undiagnosed ulcers were found in the anterior and posterior wall of the hypopharynx, the positive cells of SARS-CoV-2 were found in the whole pharyngeal wall from the mucosa of the ulcer to the deep muscle layer, and the SARS-CoV-2 RNA was detected by molecular biological techniques. Histopathology, immunohistochemistry, and molecular biology confirmed that it was a local ulcerative injury caused by SARS-CoV-2 (Porzionato et al., 2021). Endoscopic evaluation was performed on six COVID-19 patients with gastrointestinal symptoms, one severe patient had an ulcer with a diameter of about 4–6 mm in his esophagus, and the SARS-CoV-2 RNA was detected in his stomach, duodenum and rectum, but no SARS-CoV-2 RNA in the other three non-severe COVID-19 patients. It was suspected that the occurrence of ulcers was directly related to SARS-CoV-2 infection, and was also related to the disease degree of the patients (Lin et al., 2020). Deb et al. (2021) found that three COVID-19 patients had large, deep, and more than one non-hemorrhagic gastric ulcer, all resulting in death due to poor prognosis. Taherifard et al. (2022) reviewed a case of gastric ulcer induced by cytomegalovirus(CMV) after SARS-CoV-2 infection, and listed a number of similar cases, which were suspected to be further caused by the immune disorder of patients after SARS-CoV-2 infection. Of the seven COVID-19 patients, one was found to have ulcerative and ischemic changes by enterosigmoidoscopy (Seeliger et al., 2020). Therefore, gastrointestinal ulcerative lesions were more likely to appear in the later stage of SARS-CoV-2 infection, and patients’ clinical manifestations should be monitored more closely to make a timely diagnosis and treatment.

### 2.3. Gastrointestinal bleeding

Gastrointestinal bleeding often occurs in critically ill patients with COVID-19; according to statistics, the proportion of gastrointestinal bleeding in COVID-19 patients varies from 2 to 3%, which is related to the number of cases included in the statistics and clinical symptoms. Barrett et al. (2020) found that six COVID-19 patients were accompanied by gastrointestinal bleeding, manifested as blood in the stool or black stool, left lower limb ischemia, and only dyspnea, and the bleeding symptoms appeared simultaneously with typical symptoms of COVID-19. Gulen and Satar (2020) reported a case of gastrointestinal bleeding in a COVID-19 patient with dyspnea and abdominal pain accompanied by diarrhea, which was thought to be related to the drug action and SARS-CoV-2 infection. The causes of gastrointestinal bleeding in COVID-19 patients are summarized as follows: Firstly, gastrointestinal ulcer and inflammation can cause gastrointestinal bleeding; Secondly, the direct damage of SARS-CoV-2 to the mucous membranes; Thirdly, some anticoagulant or anti-inflammatory drugs can also cause gastrointestinal bleeding.

### 2.4. Gastrointestinal thrombotic lesions

The excessive expression of inflammatory factors in COVID-19 patients can also cause abnormal coagulation and thrombotic diseases. Studies have found that severe COVID-19 patients had longer prothrombin time and higher plasma D-dimer levels, which preliminarily indicated that the coagulation function of patients was disordered. In addition, the levels of tumor necrosis factor-α (TNF-α), interleukin -1β (IL-1β), and IL-8 in the plasma of patients were higher than those of ordinary people (Huang et al., 2020), and these inflammatory factors could regulate the transformation of coagulation process to the coagulation-promoting direction, and even led to thrombosis. Three cases of coagulopathy have been reported in patients with COVID-19, all of which showed an association of COVID-19 with hypercoagulability and thrombotic disease, and the symptom was also manifested in the gastrointestinal disease (Zhang et al., 2020). For example, intestinal obstruction, intestinal ischemia, acute mesenteric thrombosis, gas accumulation, and even colon perforation in patients with COVID-19 were all associated with the hypercoagulability caused by COVID-19.

Ibrahim et al. (2020) reported two cases of SARS-CoV-2 infection complicated with paralytic ileus. The first patient suffered from abdominal distension, upper abdominal tenderness, excessive bowel sounds, rectal cavity, and other symptoms after admission, the extensive large intestine dilatation and the perforation of the middle transverse colon were found during the operation. The second patient was admitted to the hospital with abdominal pain, and an abdominal X-ray showed the diffuse dilatation of the small intestine and large intestine loop, which was thought to be associated with SARS-COV-2-induced microthrombosis. Another 13-year-old male patient had small intestinal obstruction when he was admitted to the hospital, and the detection of SARS-CoV-2 reverse transcription-polymerase chain reaction (RT-PCR) was negative. As the disease progressed, the patient successively showed typical symptoms of COVID-19 respiratory system, heart, and gastrointestinal involvement. The patient later tested positive for SARS-CoV-2 immunoglobulin G (IgG) antibody, and the series of reactions showed late COVID-19 (Alsabri et al., 2020). The above results showed that intestinal obstruction could occur both before and after respiratory symptoms of COVID-19. In addition, if the colonic obstruction caused by SARS-CoV-2 infection is not found and treated in time, it may further develop into colonic ischemia and perforation.

Chan et al. (2020) reported the first case of ischemic colitis in a hypercoagulable patient, which was accompanied by abdominal pain, bloody diarrhea, and hyperactivity of bowel sounds, consistent with the clinical manifestations of ischemic colitis. Moreover, during the onset of the disease, the high inflammation and cytokine storms related to COVID-19 appeared simultaneously, both in the second week of the SARS-CoV-2 infection. At which time the inflammation and hypercoagulable state were pronounced, and the level of D-dimer was significantly increased. It was reasonable to believe that the occurrence of ischemic colitis was related to SARS-CoV-2 infection. Almeida Vargas et al. (2020) reported that all three COVID-19 patients suffered from severe colonic ischemia, and all the levels of D-dimer were significantly increased, they were attributed to the hypercoagulable state and disseminated intravascular coagulation associated with SARS-CoV-2. All three patients died shortly after diagnosis. A male patient was diagnosed with COVID-19 after presenting with a cough and shortness of breath. On the 11th day of admission, an abdominal CT scan revealed intestinal wall necrosis in the mucosa of the small intestine, and histopathological examination revealed thrombus formation in the mesenteric vessels and extensive ischemia. However, the patient’s medical history showed no hypercoagulability, venous thrombotic disease, or malignant tumor, indicating that there was no risk factor for thrombotic mesenteric ischemia, which was ultimately considered a complication of COVID-19 (Sehhat et al., 2020). There were also cases of gastrointestinal tract involvement without respiratory symptoms, four patients with COVID-19 were negative for nucleic acid, three of whom had signs of acute intestinal ischemia, and another was diagnosed with small intestinal ischemia, and SARS-CoV-2 positive was detected in intestinal resections, and levels of D-dimer in three of the four patients were significantly higher than average value (Norsa et al., 2020; Zamboni et al., 2022). That is to say, SARS-CoV-2 infection should also be considered in the presence of gastrointestinal symptoms, such as intestinal ischemia, in the absence of respiratory symptoms. In addition, Cheung S. et al. (2020) reported a COVID-19 patient without major factors for thromboembolism formation, who presented signs of acute mesenteric thrombosis and intestinal ischemia 1 week after turning negative, but the level of D-dimer was also significantly increased, which was suspected to be a potential complication caused by COVID-19. A patient with severe abdominal pain was diagnosed with COVID-19, the CT scan showed the possibility of arterial thrombosis in the mesentery, and his condition improved after the operation and taking anticoagulants. Another suspected COVID-19 case was found to have intestinal obstruction with fever, the CT scan showed portal vein and mesenteric vein thrombosis, mesenteric abdominal and pelvic effusion, followed by jejunal perforation and peritonitis, and eventually died. The levels of D-dimer in both cases were also abnormal (Rodriguez-Nakamura et al., 2020). The above research results indicated that the gastrointestinal diseases, such as intestinal ischemia and intestinal thrombosis, would appear before respiratory symptoms or secondary to SARS-CoV-2 infection, which were related to a blood hypercoagulable state caused by SARS-CoV-2. D-dimer levels in patients with COVID-19 can be measured clinically. When the level was significantly elevated, intestinal ischemia or intestinal thrombosis should be closely watched.

Intestinal ischemia and obstructive gastrointestinal diseases are usually associated with secondary pneumatosis, and multiple cases of COVID-19 patients have been found to be complicated by intestinal gas. Meini et al. (2020) reported a male patient, who was diagnosed with COVID-19 due to fever, cough, chest pain, and other symptoms, and the patient recovered completely after treatment. However, the level of D-dimer in the patient increased, and the abdominal CE-CT scan showed the presence of air in the cecum and right hemicolon, which was compatible with pneumatosis intestinalis (PI). He recovered completely after treatment, it was considered that the pneumatosis was related to the damage to the intestinal wall and intestinal microorganisms by SARS-CoV-2. To sum up the other reported case types, including one case presented with diffuse abdominal pain and rectal bleeding, and gas accumulation in the middle part of ascending colon, one transplant patient receiving immunosuppressive drugs was also infected with COVID-19 and complicated with the PI, extensive pneumatosis was detected in the small and large intestine in one case, two cases had free air in the abdominal cavity, and one case had obvious pneumatosis in the portal vein. All the above reports have fully demonstrated that the PI has become a typical complication of COVID-19 (Aiello et al., 2020; Kielty et al., 2020; Lakshmanan and Toubia, 2021). Furthermore, Bhayana et al. (2020) observed intestinal pneumatosis or portal pneumatosis in the abdominal CT of four patients with COVID-19, and three had apparent intestinal infarction during laparotomy, which also indicated the correlation between SARS-CoV-2 infection and pneumatosis intestinalis, intestinal obstruction, and other diseases.

In addition, with the continuous epidemic of COVID-19, an increasing number of case reports have found that patients also presented with gastrointestinal perforation, mainly as a CT diagnosis showing segmental intestinal wall thickening, focal intestinal wall defects, or gas bubbles outside the lumen concentrated near the intestinal wall, which was considered to be related to the direct effect of SARS-CoV-2 infection, the direct inflammation of vascular endothelium caused by microvascular thrombosis, mesenteric ischemia and intestinal obstruction (Pan Y. et al., 2020). Giuffrè et al. (2020) reported the first case of rectal perforation caused by SARS-CoV-2 infection, the patient was admitted to the hospital due to cough, abdominal pain, and high fever, and the abdominal CT showed rectal perforation, which was suspected to be a complication by COVID-19. Al Argan et al. (2021) reported that three patients with COVID-19 had a gastrointestinal perforation in different degrees. Case 1 presented abdominal pain and tenderness upon treatment, and case 2 showed anemia and peritoneal hematoma with retroperitoneal hematoma, all of which were different manifestations of gastrointestinal perforation caused by COVID-19, and it was found that in different periods of the disease course. This indicated that gastrointestinal perforation might occur at any time during SARS-CoV-2 infection. One patient with COVID-19 was reported to have colon perforation causing gastrointestinal ischemia, which was suspected to be related to sepsis and thromboembolism following SARS-CoV-2 infection (Neto et al., 2020). Another report found that a patient with COVID-19 first presented with diarrhea, then developed abdominal pain and obvious abdominal distension. The X-ray plain film and CT scan found colon perforation, as well as a tiny perforation of the anterolateral cecum (De Nardi et al., 2020). Histopathology showed that acute inflammation, necrosis, and bleeding were found in the resected intestinal specimens of COVID-19 patients with transverse colon perforation, indicating that COVID-19-induced microthrombosis led to perforation (Huang et al., 2020).

The above results showed that SARS-CoV-2 infection was likely to increase the risk of gastrointestinal thrombotic events and related ischemia, and might lead to the occurrence of other gastrointestinal diseases, resulting in increased mortality. In clinical treatment, if patients have acute abdominal pain and hematochezia, thrombosis and ischemic events should be considered in time, and the corresponding anticoagulant drugs should be used to prevent and treat such gastrointestinal thrombosis-related diseases.

## 3. Mechanism of gastrointestinal injury induced by SARS-CoV-2

**Figure 1:**
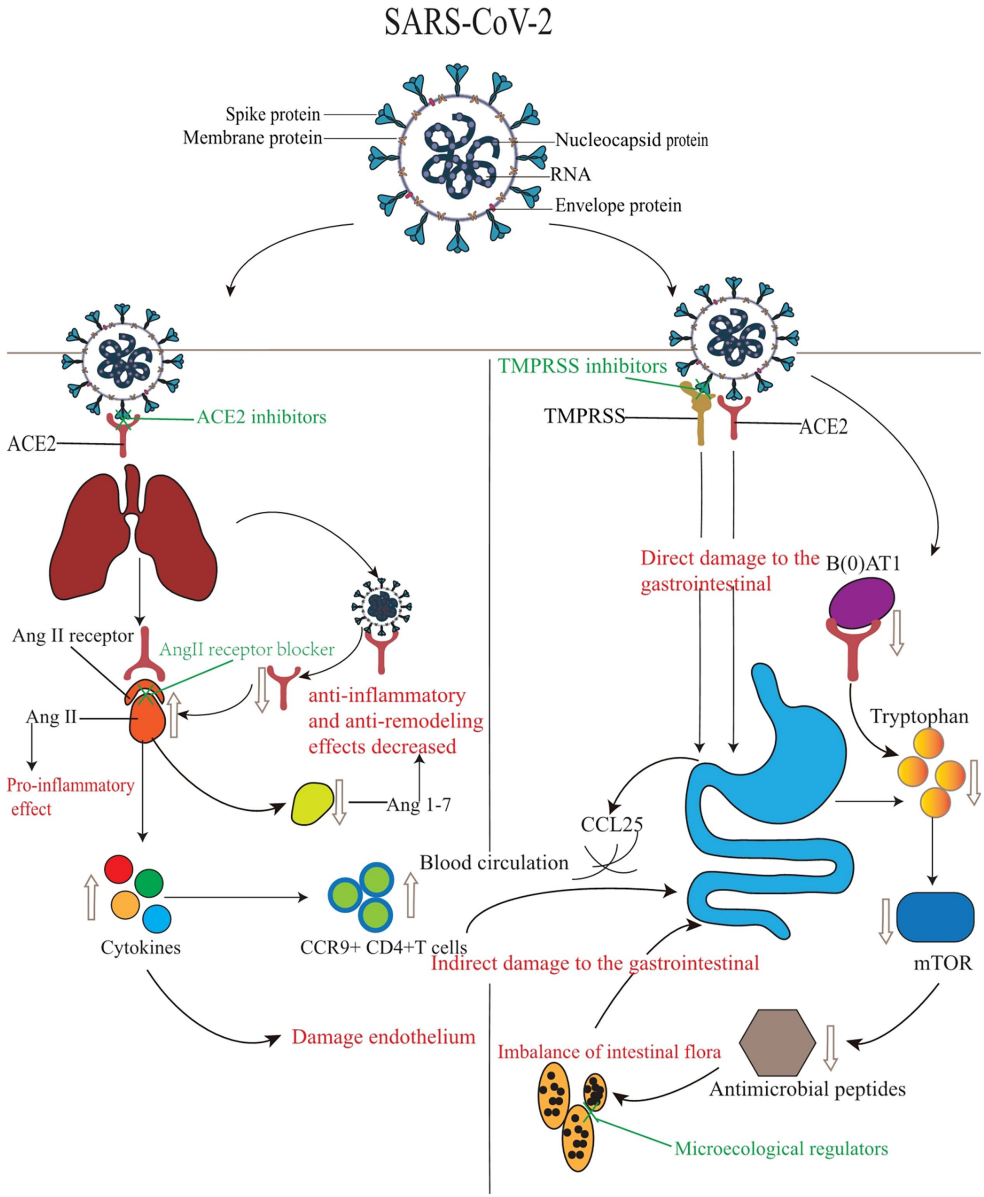
The mechanisms and treatment of gastrointestinal injury induced by SARS-CoV-2.

The pathogenesis of gastrointestinal diseases caused directly or indirectly by SARS-CoV-2 infection can be divided into the following categories (Figure 1):

The injury mechanisms of SARS-CoV-2 to the gastrointestinal tract are mainly divided into direct injury and indirect injury. The left side shows indirect injury. SARS-CoV-2 binds to the lung ACE2 receptor, and the expression of ACE2 protein is down-regulated, which affects the transformation of Ang 1–7, increases the concentration of AngII and cytokines, and leads to the disorder of RAS system and the decrease of intestinal mucosal anti-inflammatory ability. In addition to directly damaging endothelial cells, cytokines also promote lung-derived CCR9+ CD4 + T cells to enter the small intestine through the circulation mediated by CCL25 to achieve lung-intestinal axis infection. The right side shows direct damage. In addition to ACE2, serine protease also participates in the infection process through interaction with the virus S protein, which jointly damages the gastrointestinal tract. In addition, the expression of ACE2 protein is down-regulated, and the expression of the sodium-dependent neutral amino acid transporter B(0)AT1 is decreased, resulting in a significant decrease in the level of tryptophan, affecting the activity of the mTOR pathway, reducing the expression of antimicrobial peptides, and leading to local enteritis and diarrhea. In the figure, the red font represents the corresponding phenomenon or disease caused by SARS-CoV-2, while the green font represents the therapeutic measures or drugs used for the corresponding target. Figure 1 is cited from Ye Q. et al. (2020).

### 3.1. ACE2 receptor mechanism

SARS-CoV-2 infection mainly depends on the S pro on its surface. ACE2 is the primary receptor of SARS-CoV-2, which can form a SARS-CoV-2-ACE2 complex with SARS-CoV-2, then the membrane fusion is initiated, and the RNA is released into the cytoplasm of the host cell to complete the infection process (Wrapp et al., 2020; V’kovski et al., 2021; Jackson et al., 2022). It has been found that the ACE2 expression in the epithelial cells of the stomach, duodenum, ileum, and rectum was 100 times higher than that in the lung (Lamers et al., 2020; Liang et al., 2020; Xiao et al., 2020), which fully suggested that SARS-CoV-2 could infect the gastrointestinal tract by directly binding to the ACE2 receptor in the gastrointestinal tract, thereby inducing damage, inflammation and related dysfunction of the gastrointestinal epithelium and mucosa, and leading to the inflammatory lesions of the gastrointestinal tract.

### 3.2. Affect the renal angiotensinogen system (RAS)

ACE2 also plays an essential role in the regulation of the RAS. As the primary bioactive molecule of the RAS, angiotensinogen II (AngII) can be transformed into angiotensin 1–7 (Ang 1–7) by ACE2, which plays an anti-inflammatory and anti-remodeling role (Santos et al., 2018; Paz Ocaranza et al., 2020). ACE2 also plays an essential role in the regulation of the RAS. As the primary bioactive molecule of the RAS, angiotensinogen II (AngII) can be transformed into angiotensin 1–7 (Ang 1–7) by ACE2, which plays an anti-inflammatory and anti-remodeling role. However, the SARS-CoV-2-ACE2 complex was formed in the host after SARS-CoV-2 infection, which led to the down-regulation of ACE2 protein expression and affected the transformation of Ang 1–7, thus leading to the disorder of the RAS and the decrease of anti-inflammatory ability of intestinal mucosa, and further causing intestinal inflammatory symptoms in patients infected with SARS-CoV-2 (Zhang et al., 2021). Some studies have shown that the plasma Ang II levels had been found to be significantly higher in patients with COVID-19 than in those without infection (Liu Y. et al., 2020). When the expression of ACE2 was down-regulated, the concentration of Ang II increased, which had a pro-inflammatory effect and could lead to the increase of a series of cytokines in the blood, causing the endothelium damage, and making the blood to be in a hypercoagulable state, thus inducing the gastrointestinal coagulation disease (Eguchi et al., 2018). In addition, SARS-CoV-2 infection can also generate gastrointestinal coagulopathy by directly damaging the vascular endothelium (Jose and Manuel, 2020; Pamukçu, 2020; Xiao et al., 2020).

### 3.3. Affect the stability of intestinal flora

ACE2 was also an essential regulator for the maintenance of intestinal homeostasis and the stability of gut microbiota (Yoshida et al., 2020), and its knockout can alter the gut microbiota, thereby inducing colitis. At the same time, aberrant intestinal flora has also been suggested to be a major contributing factor in the pathogenesis of ulcerative lesions (Hashimoto et al., 2012; Ghoshal et al., 2020). Significant changes have been found in the intestinal microflora of SARS-CoV-2 patients during the infection, which may lead to a decrease in symbiotic bacteria with critical physiological functions, thus triggering the disorders of the intestinal microflora (Mazza et al., 2020; Hazan et al., 2022). ACE2 affected the composition of the gut microbiota by regulating the expression of neutral amino acid transporters in the gut. Studies have shown that the expression of the sodium-dependent neutral amino acid transporter B(0)AT1 was dependent on the presence of ACE2, and tryptophan was mainly absorbed through B(0)AT1/ACE2 transport pathway, and the tryptophan levels were significantly reduced when B(0)AT1 was knocked out in mice, affecting the activity of the Sirolimus (mTOR) pathway, which affected the expression of antimicrobial peptides that regulated the gut microbiota, which was necessary for the ecological balance of the gut microbiota, and its imbalance can lead to local enteritis and diarrhea, etc. (Bröer et al., 2011; Hashimoto et al., 2012; Singer et al., 2012). Several biosynthetic pathways, including the tryptophan biosynthetic pathway, have been found to be significantly altered in patients with COVID-19, possibly by gut microbes and depletion of several microbial-derived metabolites in the feces (Yeoh et al., 2021; Zuo et al., 2021); It is closely associated with modulating the inflammatory response of the host and promoting tolerance and resistance to viral pathogens (Catanzaro et al., 2020; McIlroy et al., 2020), and it has been speculated that SARS-CoV-2 may cause gastrointestinal symptoms by affecting gut microbiota stability. Furthermore studies have found that when the S1 subunit of SARS-CoV-2 S protein bound to ACE2, the B(0)AT1/ACE2 complex would be internalized in the intestine, which also affected the release of intestinal proinsulin and further affected the stability of intestinal flora (Yan et al., 2020).

### 3.4. Serine proteases mechanism

In addition to ACE2, SARS-CoV-2 infected cells also interacted with the S protein of SARS-CoV-2, such as serine protease 2 (TMPRSS2) and serine protease 4 (TMPRSS4), which were involved in the serine proteases process (Wruck and Adjaye, 2020; Zang et al., 2020). Among them, the TMPRSS2 was highly expressed in the intestinal epithelial cells (IEC), thereby increasing the susceptibility of gastrointestinal tissues to SARS-CoV-2 infection (Ding and Liang, 2020). Zang et al. (2020) Investigated the effect of TMPRSS4 on SARS-CoV-2 S protein-mediated intercellular fusion, and found that the TMPRSS4 could activate the S protein of SARS-CoV-2 and enhance the membrane fusion, and play a cis-like role in promoting SARS-CoV-2 infection in the intestine. Treatment of SARS-CoV-2 with TMPRSS inhibitor showed that it could significantly inhibit SARS-CoV-2 infection. Furthermore, Lee et al. (2020) investigated their role in SARS-CoV-2 infection of the gastrointestinal tract in human intestinal cell lines containing high levels of TMPRSS2 and ACE2, and found that these cells were able to infect SARS-CoV-2 persistently, and had a robust transmission. These results indicated that serine protease played an important role in mediating SARS-CoV-2 infection of gastrointestinal cells. For gastrointestinal infections, studies have suggested that targeting TMPRSS2 and TMPRSS4 may reduce the chance of SARS-CoV-2 infection (Zang et al., 2020). For example, camostat mesylate (NI-03), a TMPRSS2 inhibitor, was found to inhibit SARS-CoV-2 infection in human lung cells. Hoffmann et al. (2020a,b) found that nafamostat mesylate was significantly more effective than camostat mesylate in blocking SARS-CoV-2 infection in human lung cells, and its safety was also confirmed. Therefore, serine protease inhibitors should also be considered for the treatment of COVID-19.

### 3.5. Gastrointestinal injury through the lung-gut axis

In addition to the inflammatory reaction and flora imbalance caused by direct infection of the gastrointestinal cells, lung cells infected by SARS-CoV-2 can also cause gastrointestinal injury and gastrointestinal symptoms. The gastrointestinal flora affected the respiratory tract through the mucosal immune system, and the respiratory flora could also affect the digestive tract through immunomodulation (Wang et al., 2018; Domínguez-Díaz et al., 2019). The C-C chemokine type 9 receptor was an essential chemokine receptor for CD4 + T cells to enter the small intestine. Some studies have found that lung-derived CCR9 + CD4 + T cells would be increased after infection, and the small intestinal epithelium could express CCL25, which could promote CCR9 + CD4 + T cells to enter the small intestine, and CD4 + T cells to enter the small intestine would lead to intestinal immune injury, causing a series of mucosal reactions and chronic enteritis (Stenstad et al., 2006; Wang et al., 2014). Therefore, it was very likely that the gastrointestinal diseases were caused by the imbalance of respiratory flora after the SARS-CoV-2 infection. In addition, after pulmonary infection, ACE2 receptors made it easy for vessels in the lungs to become target organs, triggering microthrombosis, immune complex deposition, and excessive immune responses. However, it was highly likely that SARS-CoV-2 particles reached the intestinal epithelium under the protection of the mucus layer for infection, resulting in local gut inflammation and secondary intestinal injury (Garg et al., 2020; Kumar et al., 2020).

## 4. Treatment

COVID-19 patients may also suffer from a series of gastrointestinal symptoms due to related drugs during treatment (Figure 1). For example, Remdesivir is used to treat COVID-19 patients, patients usually show elevated levels of transaminase or bilirubin, anorexia, nausea, vomiting, diarrhea and other gastrointestinal symptoms (Cao et al., 2020; Spinner et al., 2020; Wang Y. et al., 2020). For Lopinavir/Ritonavir combination therapy, gastrointestinal adverse events are more likely to occur, including nausea, diarrhea and elevated liver enzymes. Studies have shown that about 8.1% of patients will develop grade 2–3 gastrointestinal disease and discontinue medication (Cao et al., 2020; Ye X. T. et al., 2020; Lu et al., 2021). In addition, because chloroquine phosphate alone has not shown a benefit in the treatment of hospitalized patients with COVID-19 and increases mortality, its potential risks cannot be predicted. It is not recommended for use alone in patients with COVID-19 (Llover and Jiménez, 2021; Singh et al., 2021). Other studies have shown that treatment with some proprietary Chinese medicines can also cause elevated serum transaminase, diarrhea, stomach pain, nausea and vomiting and other gastrointestinal symptoms (Duan et al., 2020; Zhang et al., 2020). Therefore, it is crucial to learn to identify clinically whether a patient has a gastrointestinal disorder due to the effects of a drug. SARS-CoV-2 enters cells by binding to ACE2, therefore, the gastrointestinal symptoms of patients with COVID-19 can be regulated by blocking the binding of SARS-CoV-2 to ACE2. It has been found that ACE2 inhibitors can regulate intestinal amino acid metabolism, secretion of antimicrobial peptides, intestinal microbial stability, and innate immunity (Li et al., 2020). azathioprine, for example, has been found to compete with ACE2 for binding regions or act as an ACE2 inhibitor (Chen et al., 2020). Merarchi et al. (2021) found that baicalin, hesperidin and glycyrrhizin could act as ACE2 inhibitors. The novel antibody combination IBI314 can bind to two different RBDS, block the interaction between RBD and ACE2 receptor, and show the super-potent neutralization effect on SARS-CoV-2, and can also act as an ACE2 inhibitor (Zou et al., 2022). Thus, ACE2 inhibitors can be used to treat the gastrointestinal diseases caused by SARS-CoV-2. In addition, the increase of AngII has a pro-inflammatory effect, which can damage the vascular endothelial cells and induce gastrointestinal coagulation disease. Therefore, AngII receptor blockers can also be used as therapeutic drugs for gastrointestinal diseases caused by SARS-CoV-2 infection, and appropriate anticoagulants can also be used in advance for prevention in clinical treatment. However, whether SARS-CoV-2 infects the gastrointestinal directly or indirectly, the microflora will be affected and unbalanced in most cases. A healthy gut microbiota can control SARS-CoV-2-induced lung infection by producing large numbers of immune cells. For example, probiotics and prebiotics in the diet can inhibit SARS-CoV-2 infection by regulating the homeostasis of the gut microbiota, and their effectiveness has been demonstrated in multiple studies and clinical trials (d’Ettorre et al., 2020; de Ponte et al., 2021; Rajput et al., 2021). Microecological modulators also maintain intestinal mucosal integrity and minimize secondary bacterial infections (Wei, 2020). Therefore, regulation of the stability of the gut microbiota and maintenance of the integrity of the intestinal mucosa may also be an alternative treatment for gastrointestinal diseases caused by SARS-CoV-2 infection. Targeting the role of serine proteases in gastrointestinal disease caused by SARS-CoV-2 infection, inhibiting serine proteases may also be a therapeutic option. So far, several studies have reported the effect of inhibiting TMPRSS2 on the infection of SARS-CoV-2 in the lungs (Hoffmann et al., 2020a,b). There are two pathways: one is the direct inhibition of TMPRSS2 activity by drug inhibitors. The other is to inhibit TMPRSS2 activity through chemical interactions with corresponding residues, such as ASP435, SER441, and His296, all of which are critical for the downregulation of TMPRSS2 expression (Ko et al., 2020). However, a variety of drugs currently in clinical trials, such as Bromohexine, Ambroxol, Carmustat, and Binaphrostat, have side effects to varying degrees (Breining et al., 2021; Hoffmann et al., 2021; Kehinde et al., 2022). It may be possible to use the affinity of natural compounds to bind different amino acid residues of TMPRSS2 to inhibit TMPRSS2. Such natural drugs need to be further searched and studied. Moreover, due to the presence of the lung-gut axis, the gastrointestinal involvement may also be caused by pulmonary infection. Therefore, inhibition of gastrointestinal injury by SARS-CoV-2 may start from the inhibition of pulmonary infection, and then inhibit the infection of SARS-CoV-2 on the gastrointestinal tract by immunomodulation or regulation of respiratory flora, but it should also be considered whether the drug will aggravate the symptoms of the gastrointestinal tract of patients when using the drug.

## 5. Summary

Although the main symptoms of COVID-19 are respiratory symptoms, gastrointestinal symptoms are becoming more frequent. Many studies have reported that the patients with COVID-19 will develop or be complicated with gastrointestinal disease before respiratory diseases, after respiratory symptoms or after recovery (Cheung K. S. et al., 2020; Holshue et al., 2020; Jin et al., 2020; Lin et al., 2020; Pan L. et al., 2020). Whether the gastrointestinal disease is caused directly or indirectly by SARS-CoV-2, it is related to its receptor or enzyme. For example, ACE2 receptor and serine proteases play a critical role in the process of SARS-CoV-2 infection of cells, and the entry mechanisms of SARS-CoV-2 involve many organs such as the brain, lung, gastrointestinal tract, and liver. The infection can also be interconnected between them through the corresponding channels. The imbalance of the corresponding microbiota in the gastrointestinal tract will also impact the gastrointestinal diseases caused by SARS-CoV-2 infection, further increasing the possibility of gastrointestinal involvement. Therefore, inhibiting or blocking the corresponding receptors and enzymes, and regulating the stability of the interconnected flora in the gastrointestinal tract can produce a definite therapeutic effect on the gastrointestinal diseases caused by SARS-CoV-2 infection, however, the specific effective therapeutic drugs need further research and development. Furthermore, if COVID-19 patients with gastrointestinal symptoms caused by SARS-CoV-2 infection were treated as ordinary patients without significant respiratory symptoms, the consequences would be unimaginable. In addition, after COVID-19 patients have recovered from treatment, if measures to prevent gastrointestinal diseases are not taken in time, the likelihood of patients with poor prognosis or even death will be increased. Therefore, timely identification and understanding of the gastrointestinal symptoms, injury mechanisms, and treatment approach caused by SARS-CoV-2 infection can be beneficial for early prevention and rational treatment in clinical practice.

## Author contributions

AS conceived and planned the overall structure of the review, wrote the manuscript, and finally approved the manuscript. YL and XZ collected the references and wrote the manuscript. AS and YL collaboratively finished the revision of the manuscript. All authors contributed to the article and approved the submitted version.

## Funding

This work was supported by the Science and Technology Research Project of Chongqing Education Commission (KJQN201901235, KJQN20201226, and KJQN202101247) and the Talent Introduction and Scientific Research Startup Fund Project of Chongqing Three Gorges University (20190002).

## Conflict of interest

The authors declare that the research was conducted in the absence of any commercial or financial relationships that could be construed as a potential conflict of interest.

## Publisher’s note

All claims expressed in this article are solely those of the authors and do not necessarily represent those of their affiliated organizations, or those of the publisher, the editors and the reviewers. Any product that may be evaluated in this article, or claim that may be made by its manufacturer, is not guaranteed or endorsed by the publisher.

## References

[ref1] Aiello P. Johnson S. Ramos Mercado A. Hussein S. (2020). Pneumatosis intestinalis in a patient with COVID-19. BMJ Case Rep. 13:e237564. doi: 10.1136/bcr-2020-237564, PMID: 32900750 PMC7478032

[ref2] Al Argan R. J. Alqatari S. G. Al Said A. H. Alsulaiman R. M. Noor A. Al Sheekh L. A.. (2021). Gastrointestinal perforation secondary to COVID-19: case reports and literature review. Medicine 100:e25771. doi: 10.1097/MD.0000000000025771, PMID: 34106608 PMC8133225

[ref3] Alberca G. G. F. Solis-Castro R. L. Solis-Castro M. E. Alberca R. W. (2021). Coronavirus disease-2019 and the intestinal tract: an overview. World J. Gastroenterol. 27, 1255–1266. doi: 10.3748/wjg.v27.i13.1255, PMID: 33833480 PMC8015300

[ref4] Almeida Vargas A. Valentí V. Sánchez Justicia C. Martínez Regueira F. Martí Cruchaga P. Luján Colás J.. (2020). Severe colon ischemia in patients with severe coronavirus-19 (COVID-19). Rev. Esp. Enferm. Dig. 112, 784–787. doi: 10.17235/reed.2020.7329/2020, PMID: 32954769

[ref5] Alsabri M. Sakr M. Qarooni S. Hassanein M. M. (2020). COVID-19 infection in a child presenting with functional intestinal obstruction. Cureus 12:e11448. doi: 10.7759/cureus.11448, PMID: 33329947 PMC7733780

[ref6] Bacaksız F. Ebik B. Ekin N. Kılıc J. (2021). Pancreatic damage in COVID-19: why? How? Int. J. Clin. Pract. 75:e14692. doi: 10.1111/ijcp.14692, PMID: 34331821 PMC8420122

[ref7] Barrett L. F. Lo K. B. Stanek S. R. Walter J. W. (2020). Self-limited gastrointestinal bleeding in COVID-19. Clin. Res. Hepatol. Gastroenterol. 44, e77–e80. doi: 10.1016/j.clinre.2020.06.015, PMID: 32753264 PMC7362869

[ref8] Bhayana R. Som A. Li M. D. Carey D. E. Anderson M. A. Blake M. A.. (2020). Abdominal imaging findings in COVID-19: preliminary observations. Radiology 297, E207–E215. doi: 10.1148/radiol.2020201908, PMID: 32391742 PMC7508000

[ref9] Breining P. Frølund A. L. Højen J. F. Gunst J. D. Staerke N. B. Saedder E.. (2021). Camostat mesylate against SARS-CoV-2 and COVID-19-rationale, dosing and safety. Basic Clin. Pharmacol. Toxicol. 128, 204–212. doi: 10.1111/bcpt.13533 33176395

[ref10] Bröer A. Juelich T. Vanslambrouck J. M. Tietze N. Solomon P. S. Holst J.. (2011). Impaired nutrient signaling and body weight control in a Na+ neutral amino acid cotransporter (Slc6a19)-deficient mouse. J. Biol. Chem. 286, 26638–26651. doi: 10.1074/jbc.M111.241323, PMID: 21636576 PMC3143628

[ref11] Bruno G. Fabrizio C. Santoro C. R. Buccoliero G. B. (2021). Pancreatic injury in the course of coronavirus disease 2019: a not-so-rare occurrence. J. Med. Virol. 93, 74–75. doi: 10.1002/jmv.26134, PMID: 32497298 PMC7300736

[ref12] Calabrese E. Zorzi F. Monteleone G. Del Vecchio Blanco G. (2020). Onset of ulcerative colitis during SARS-CoV-2 infection. Dig. Liver Dis. 52, 1228–1229. doi: 10.1016/j.dld.2020.06.003, PMID: 32601030 PMC7287422

[ref13] Cao B. Wang Y. Wen D. Liu W. Wang J. Fan G.. (2020). A trial of lopinavir-ritonavir in adults hospitalized with severe COVID-19. N. Engl. J. Med. 382, 1787–1799. doi: 10.1056/NEJMoa2001282 32187464 PMC7121492

[ref14] Carvalho A. Alqusairi R. Adams A. Paul M. Kothari N. Peters S.. (2020). SARS-CoV-2 gastrointestinal infection causing hemorrhagic colitis: implications for detection and transmission of COVID-19 disease. Am. J. Gastroenterol. 115, 942–946. doi: 10.14309/ajg.0000000000000667, PMID: 32496741 PMC7172485

[ref15] Catanzaro M. Fagiani F. Racchi M. Corsini E. Govoni S. Lanni C. (2020). Immune response in COVID-19: addressing a pharmacological challenge by targeting pathways triggered by SARS-CoV-2. Signal Transduct. Target. Ther. 5:84. doi: 10.1038/s41392-020-0191-1, PMID: 32467561 PMC7255975

[ref16] Chan K. H. Lim S. L. Damati A. Maruboyina S. P. Bondili L. Abu Hanoud A.. (2020). Coronavirus disease 2019 (COVID-19) and ischemic colitis: an under-recognized complication. Am. J. Emerg. Med. 38:2758.e1. doi: 10.1016/j.ajem.2020.05.072, PMID: 32499176 PMC7251350

[ref17] Chen Y. Guo Y. Pan Y. Zhao Z. J. (2020). Structure analysis of the receptor binding of 2019-nCoV. Biochem. Biophys. Res. Commun. 525, 135–140. doi: 10.1016/j.bbrc.2020.02.071, PMID: 32081428 PMC7092824

[ref18] Chen T. H. Hsu M. T. Lee M. Y. Chou C. K. (2022). Gastrointestinal involvement in SARS-CoV-2 infection. Viruses 14:1188. doi: 10.3390/v14061188, PMID: 35746659 PMC9228950

[ref19] Cheung K. S. Hung I. F. N. Chan P. P. Y. Lung K. C. Tso E. Liu R.. (2020). Gastrointestinal manifestations of SARS-CoV-2 infection and virus load in fecal samples from a Hong Kong cohort: systematic review and meta-analysis. Gastroenterology 159, 81–95. doi: 10.1053/j.gastro.2020.03.065, PMID: 32251668 PMC7194936

[ref20] Cheung S. Quiwa J. C. Pillai A. Onwu C. Tharayil Z. J. Gupta R. (2020). Superior mesenteric artery thrombosis and acute intestinal ischemia as a consequence of COVID-19 infection. Am. J. Case Rep. 21:e925753. doi: 10.12659/AJCR.925753, PMID: 32724028 PMC7417027

[ref21] De Nardi P. Parolini D. C. Ripa M. Racca S. Rosati R. (2020). Bowel perforation in a COVID-19 patient: case report. Int. J. Color. Dis. 35, 1797–1800. doi: 10.1007/s00384-020-03627-6, PMID: 32458395 PMC7250585

[ref22] de Ponte M. C. Cardoso V. G. Gonçalves G. L. Costa-Pessoa J. M. Oliveira-Souza M. (2021). Early type 1 diabetes aggravates renal ischemia/reperfusion-induced acute kidney injury. Sci. Rep. 11:19028. doi: 10.1038/s41598-021-97839-7, PMID: 34561469 PMC8463569

[ref23] Deb A. Thongtan T. Costilla V. (2021). Gastric ulcerations in COVID-19: an ominous sign? BMJ Case Rep. 14:e244059. doi: 10.1136/bcr-2021-244059, PMID: 34281947 PMC8291328

[ref24] d’Ettorre G. Ceccarelli G. Marazzato M. Campagna G. Pinacchio C. Alessandri F.. (2020). Challenges in the management of SARS-CoV2 infection: the role of oral bacteriotherapy as complementary therapeutic strategy to avoid the progression of COVID-19. Front. Med. 7:389. doi: 10.3389/fmed.2020.00389, PMID: 32733907 PMC7358304

[ref25] Ding S. Liang T. J. (2020). Is SARS-CoV-2 also an enteric pathogen with potential fecal-Oral transmission? A COVID-19 virological and clinical review. Gastroenterology 159, 53–61. doi: 10.1053/j.gastro.2020.04.052, PMID: 32353371 PMC7184994

[ref26] Domínguez-Díaz C. García-Orozco A. Riera-Leal A. Padilla-Arellano J. R. Fafutis-Morris M. (2019). Microbiota and its role on viral evasion: is it with us or against us? Front. Cell. Infect. Microbio. 9:256. doi: 10.3389/fcimb.2019.00256, PMID: 31380299 PMC6657001

[ref27] Duan C. Xia W. G. Zheng C. J. Sun G. B. Li Z. L. Li Q. L.. (2020). Clinical observation of Jinhua Qinggan granule combined with conventional western medicine in the treatment of mild novel coronavirus pneumonia. J. Tradit. Chin. Med. 61, 1473–1477. doi: 10.13288/j.11-2166/r.2020.17.001

[ref28] Eguchi S. Kawai T. Scalia R. Rizzo V. (2018). Understanding angiotensin II type 1 receptor signaling in vascular pathophysiology. Hypertension 71, 804–810. doi: 10.1161/HYPERTENSIONAHA.118.10266, PMID: 29581215 PMC5897153

[ref29] Garg S. Garg M. Prabhakar N. Malhotra P. Agarwal R. (2020). Unraveling the mystery of COVID-19 cytokine storm: from skin to organ systems. Dermatol. Ther. 33:e13859. doi: 10.1111/dth.13859, PMID: 32559324 PMC7323083

[ref30] Ghoshal U. C. Ghoshal U. Mathur A. Singh R. K. Nath A. Garg A.. (2020). The spectrum of gastrointestinal symptoms in patients with coronavirus disease-19: predictors, relationship with disease severity, and outcome. Clin. Transl. Gastroenterol. 11:e00259. doi: 10.14309/ctg.0000000000000259, PMID: 33463978 PMC7678797

[ref31] Giuffrè M. Bozzato A. M. Di Bella S. Occhipinti A. A. Martingano P. Cavallaro M. F. M.. (2020). Spontaneous rectal perforation in a patient with SARS-CoV-2 infection. J. Pers. Med. 10:157. doi: 10.3390/jpm10040157 33049924 PMC7712943

[ref32] Gulen M. Satar S. (2020). Uncommon presentation of COVID-19: gastrointestinal bleeding. Clin. Res. Hepatol. Gastroenterol. 44, e72–e76. doi: 10.1016/j.clinre.2020.05.001, PMID: 32505730 PMC7241390

[ref33] Hashimoto T. Perlot T. Rehman A. Trichereau J. Ishiguro H. Paolino M.. (2012). ACE2 links amino acid malnutrition to microbial ecology and intestinal inflammation. Nature 487, 477–481. doi: 10.1038/nature11228, PMID: 22837003 PMC7095315

[ref34] Hazan S. Stollman N. Bozkurt H. S. Dave S. Papoutsis A. J. Daniels J.. (2022). Lost microbes of COVID-19: Bifidobacterium, Faecalibacterium depletion and decreased microbiome diversity associated with SARS-CoV-2 infection severity. BMJ Open Gastroenterol. 9:e000871. doi: 10.1136/bmjgast-2022-000871, PMID: 35483736 PMC9051551

[ref35] Hoffmann M. Hofmann-Winkler H. Smith J. C. Krüger N. Arora P. Sørensen L. K.. (2021). Camostat mesylate inhibits SARS-CoV-2 activation by TMPRSS2-related proteases and its metabolite GBPA exerts antiviral activity. EBioMedicine 65:103255. doi: 10.1016/j.ebiom.2021.103255, PMID: 33676899 PMC7930809

[ref36] Hoffmann M. Kleine-Weber H. Schroeder S. Krüger N. Herrler T. Erichsen S.. (2020a). SARS-CoV-2 cell entry depends on ACE2 and TMPRSS2 and is blocked by a clinically proven protease inhibitor. Cells 181, 271–280.e8. doi: 10.1016/j.cell.2020.02.052, PMID: 32142651 PMC7102627

[ref37] Hoffmann M. Mösbauer K. Hofmann-Winkler H. Kaul A. Kleine-Weber H. Krüger N.. (2020b). Chloroquine does not inhibit infection of human lung cells with SARS-CoV-2. Nature 585, 588–590. doi: 10.1038/s41586-020-2575-3 32698190

[ref38] Holshue M. L. DeBolt C. Lindquist S. Lofy K. H. Wiesman J. Bruce H.. (2020). First case of 2019 novel coronavirus in the United States. N. Engl. J. Med. 382, 929–936. doi: 10.1056/NEJMoa2001191, PMID: 32004427 PMC7092802

[ref39] Huang C. Wang Y. Li X. Ren L. Zhao J. Hu Y.. (2020). Clinical features of patients infected with 2019 novel coronavirus in Wuhan, China. Lancet 395, 497–506. doi: 10.1016/S0140-6736(20)30183-5, PMID: 31986264 PMC7159299

[ref40] Ibrahim Y. S. Karuppasamy G. Parambil J. V. Alsoub H. Al-Shokri S. D. (2020). Case report: paralytic ileus: a potential extrapulmonary manifestation of severe COVID-19. Am. J. Trop. Med. Hyg. 103, 1600–1603. doi: 10.4269/ajtmh.20-0894, PMID: 32876011 PMC7543796

[ref41] Imperatore N. Bennato R. D'Avino A. Lombardi G. Manguso F. (2021). SARS-CoV-2 as a trigger for de novo ulcerative colitis. Inflamm. Bowel Dis. 27, e87–e88. doi: 10.1093/ibd/izab040, PMID: 33616182 PMC7928823

[ref42] Jackson C. B. Farzan M. Chen B. Choe H. (2022). Mechanisms of SARS-CoV-2 entry into cells. Nat. Rev. Mol. Cell Biol. 23, 3–20. doi: 10.1038/s41580-021-00418-x, PMID: 34611326 PMC8491763

[ref43] Jin X. Lian J. S. Hu J. H. Gao J. Zheng L. Zhang Y. M.. (2020). Epidemiological, clinical and virological characteristics of 74 cases of coronavirus-infected disease 2019 (COVID-19) with gastrointestinal symptoms. Gut 69, 1002–1009. doi: 10.1136/gutjnl-2020-320926, PMID: 32213556 PMC7133387

[ref44] Jose R. J. Manuel A. (2020). COVID-19 cytokine storm: the interplay between inflammation and coagulation. Lancet Respir. Med. 8, e46–e47. doi: 10.1016/S2213-2600(20)30216-2, PMID: 32353251 PMC7185942

[ref45] Kehinde I. A. Egbejimi A. Kaur M. Onyenaka C. Adebusuyi T. Olaleye O. A. (2022). Inhibitory mechanism of Ambroxol and Bromhexine Hydrochlorides as potent blockers of molecular interaction between SARS-CoV-2 spike protein and human angiotensin-converting Enzyme-2. J. Mol. Graph. Model. 114:108201. doi: 10.1016/j.jmgm.2022.108201, PMID: 35487151 PMC9022787

[ref46] Kielty J. Duggan W. P. O'Dwyer M. (2020). Extensive pneumatosis intestinalis and portal venous gas mimicking mesenteric ischaemia in a patient with SARS-CoV-2. Ann. R. Coll. Surg. Engl. 102, e145–e147. doi: 10.1308/rcsann.2020.0145, PMID: 32538098 PMC7388941

[ref47] Ko C. J. Hsu T. W. Wu S. R. Lan S. W. Hsiao T. F. Lin H. Y.. (2020). Inhibition of TMPRSS2 by HAI-2 reduces prostate cancer cell invasion and metastasis. Oncogene 39, 5950–5963. doi: 10.1038/s41388-020-01413-w, PMID: 32778768 PMC7416816

[ref48] Kumar A. Faiq M. A. Pareek V. Raza K. Narayan R. K. Prasoon P.. (2020). Relevance of SARS-CoV-2 related factors ACE2 and TMPRSS2 expressions in gastrointestinal tissue with pathogenesis of digestive symptoms, diabetes-associated mortality, and disease recurrence in COVID-19 patients. Med. Hypotheses 144:110271. doi: 10.1016/j.mehy.2020.110271, PMID: 33254575 PMC7487155

[ref49] Lakshmanan S. Toubia N. (2021). Pneumatosis intestinalis in COVID-19. Clin. Gastroenterol. Hepatol. 19:e99. doi: 10.1016/j.cgh.2020.05.048, PMID: 32485300 PMC7260506

[ref50] Lamers M. M. Beumer J. van der Vaart J. Knoops K. Puschhof J. Breugem T. I.. (2020). SARS-CoV-2 productively infects human gut enterocytes. Science 369, 50–54. doi: 10.1126/science.abc1669, PMID: 32358202 PMC7199907

[ref51] Lee S. Yoon G. Y. Myoung J. Kim S. J. Ahn D. G. (2020). Robust and persistent SARS-CoV-2 infection in the human intestinal brush border expressing cells. Emerg. Microbes Infec. 9, 2169–2179. doi: 10.1080/22221751.2020.1827985, PMID: 32969768 PMC7580600

[ref52] Li C. Liu P. Guo S. S. Zhao Z. G. (2020). Study on the mechanism and treatment of COVID-19, SARS and MERS with gastrointestinal symptoms. Chinese J. Digest. 40, 176–179. doi: 10.3760/cma.j.issn.0254-1432.2020.03.008

[ref53] Liang W. Feng Z. Rao S. Xiao C. Xue X. Lin Z.. (2020). Diarrhoea may be underestimated: a missing link in 2019 novel coronavirus. Gut 69, 1141–1143. doi: 10.1136/gutjnl-2020-320832, PMID: 32102928

[ref54] Lin L. Jiang X. Zhang Z. Huang S. Zhang Z. Fang Z.. (2020). Gastrointestinal symptoms of 95 cases with SARS-CoV-2 infection. Gut 69:997. doi: 10.1136/gutjnl-2020-321013, PMID: 32241899

[ref55] Liu F. Long X. Zhang B. Zhang W. Chen X. Zhang Z. (2020). ACE2 expression in pancreas may cause pancreatic damage after SARS-CoV-2 infection. Clin. Gastroenterol. Hepatol. 18, 2128–2130.e2. doi: 10.1016/j.cgh.2020.04.040, PMID: 32334082 PMC7194639

[ref56] Liu Y. Yang Y. Zhang C. Huang F. Wang F. Yuan J.. (2020). Clinical and biochemical indexes from 2019-nCoV infected patients linked to viral loads and lung injury. Sci. China Life Sci. 63, 364–374. doi: 10.1007/s11427-020-1643-8, PMID: 32048163 PMC7088566

[ref57] Llover M. N. Jiménez M. C. (2021). Estado actual de los tratamientos para la COVID-19. FMC 28, 40–56. doi: 10.1016/j.fmc.2020.10.005, PMID: 33519178 PMC7826050

[ref58] Lu J. M. Zhou A. F. Zhang X. B. Xu H. Wang X. F. Ye Q. F.. (2021). Safety and efficacy of oral lopinavir/ritonavir in pediatric patients with coronavirus disease: a nationwide comparative analysis. Eur. Rev. Med. Pharmacol. Sci. 25, 549–555. doi: 10.26355/eurrev_202101_24427, PMID: 33506948

[ref59] Marasco G. Maida M. Morreale G. C. Licata M. Renzulli M. Cremon C.. (2021). Gastrointestinal bleeding in COVID-19 patients: a systematic review with meta-analysis. Can. J. Gastroenterol. Hepatol. 2021:2534975. doi: 10.1155/2021/2534975, PMID: 34513750 PMC8429023

[ref60] Massironi S. Viganò C. Dioscoridi L. Filippi E. Pagliarulo M. Manfredi G.. (2020). Endoscopic findings in patients infected with 2019 novel coronavirus in Lombardy, Italy. Clin. Gastroenterol. Hepatol. 18, 2375–2377. doi: 10.1016/j.cgh.2020.05.045, PMID: 32480008 PMC7260560

[ref61] Mazza S. Sorce A. Peyvandi F. Vecchi M. Caprioli F. (2020). A fatal case of COVID-19 pneumonia occurring in a patient with severe acute ulcerative colitis. Gut 69, 1148–1149. doi: 10.1136/gutjnl-2020-321183, PMID: 32245909

[ref62] McIlroy J. R. Mullish B. H. Goldenberg S. D. Ianiro G. Marchesi J. R. (2020). Intestinal microbiome transfer, a novel therapeutic strategy for COVID-19 induced hyperinflammation? In reply to, ‘COVID-19: immunology and treatment options,’ Felsenstein, Herbert McNamara et al. 2020. Clin. Immunol. 218:108542. doi: 10.1016/j.clim.2020.108542 32663514 PMC7354373

[ref63] Meini S. Zini C. Passaleva M. T. Frullini A. Fusco F. Carpi R.. (2020). Pneumatosis intestinalis in COVID-19. BMJ Open Gastroenterol. 7:e000434. doi: 10.1136/bmjgast-2020-000434, PMID: 32522754 PMC7287500

[ref64] Merarchi M. Dudha N. Das B. C. Garg M. (2021). Natural products and phytochemicals as potential anti-SARS-CoV-2 drugs. Phytother. Res. 35, 5384–5396. doi: 10.1002/ptr.7151, PMID: 34132421 PMC8441929

[ref65] Neto I. J. F. Viana K. F. Silva M. B. S. Silva L. M. Oliveira G. Cecchini A. R. S.. (2020). Perforated acute abdomen in a patient with COVID-19: an atypical manifestation of the disease. J. Coloproctol. 40, 269–272. doi: 10.1016/j.jcol.2020.05.011

[ref66] Norsa L. Valle C. Morotti D. Bonaffini P. A. Indriolo A. Sonzogni A. (2020). Intestinal ischemia in the COVID-19 era. Dig. Liver Dis. 52, 1090–1091. doi: 10.1016/j.dld.2020.05.030, PMID: 32532607 PMC7283075

[ref67] Pamukçu B. (2020). Inflammation and thrombosis in patients with COVID-19: a prothrombotic and inflammatory disease caused by SARS coronavirus-2. Anatol. J. Cardiol. 24, 224–234. doi: 10.14744/AnatolJCardiol.2020.56727, PMID: 33001051 PMC7585960

[ref68] Pan L. Mu M. Yang P. Sun Y. Wang R. Yan J.. (2020). Clinical characteristics of COVID-19 patients with digestive symptoms in Hubei, China: a descriptive, cross-sectional, multicenter study. Am. J. Gastroentero. 115, 766–773. doi: 10.14309/ajg.0000000000000620, PMID: 32287140 PMC7172492

[ref69] Pan Y. Zhang D. Yang P. Poon L. L. M. Wang Q. (2020). Viral load of SARS-CoV-2 in clinical samples. Lancet Infect. Dis. 20, 411–412. doi: 10.1016/S1473-3099(20)30113-4, PMID: 32105638 PMC7128099

[ref70] Paz Ocaranza M. Riquelme J. A. García L. Jalil J. E. Chiong M. Santos R. A. S.. (2020). Counter-regulatory renin-angiotensin system in cardiovascular disease. Nat. Rev. Cardiol. 17, 116–129. doi: 10.1038/s41569-019-0244-8, PMID: 31427727 PMC7097090

[ref71] Porzionato A. Stocco E. Emmi A. Contran M. Macchi V. Riccetti S.. (2021). Hypopharyngeal ulcers in COVID-19: histopathological and virological Analyses-A Case Report. Front. Immunol. 12:676828. doi: 10.3389/fimmu.2021.676828, PMID: 34290701 PMC8287416

[ref72] Rajput S. Paliwal D. Naithani M. Kothari A. Meena K. Rana S. (2021). COVID-19 and gut microbiota: a potential connection. Indian J. Clin. Biochem. 36, 266–277. doi: 10.1007/s12291-020-00948-9, PMID: 33495676 PMC7818076

[ref73] Rodriguez-Nakamura R. M. Gonzalez-Calatayud M. Martinez Martinez A. R. (2020). Acute mesenteric thrombosis in two patients with COVID-19. Two cases report and literature review. Int. J. Surg. Case Rep. 76, 409–414. doi: 10.1016/j.ijscr.2020.10.040, PMID: 33083204 PMC7560267

[ref74] Santos R. A. S. Sampaio W. O. Alzamora A. C. Motta-Santos D. Alenina N. Bader M.. (2018). The ACE2/angiotensin-(1-7)/MAS axis of the renin-angiotensin system: focus on angiotensin-(1-7). Physiol. Rev. 98, 505–553. doi: 10.1152/physrev.00023.2016, PMID: 29351514 PMC7203574

[ref75] Seeliger B. Philouze G. Benotmane I. Mutter D. Pessaux P. (2020). Is the severe acute respiratory syndrome coronavirus 2 (SARS-CoV-2) present intraperitoneally in patients with coronavirus disease 2019 (COVID-19) infection undergoing emergency operations? Surgery 168, 220–221. doi: 10.1016/j.surg.2020.05.033, PMID: 32591139 PMC7274633

[ref76] Sehhat S. Talebzadeh H. Hakamifard A. Melali H. Shabib S. Rahmati A.. (2020). Acute mesenteric ischemia in a patient with COVID-19: a case report. Arch. Iran. Med. 23, 639–643. doi: 10.34172/aim.2020.77, PMID: 32979913

[ref77] Singer D. Camargo S. M. Ramadan T. Schäfer M. Mariotta L. Herzog B.. (2012). Defective intestinal amino acid absorption in Ace2 null mice. Am. J. Physiol. Gastrointest. Liver Physiol. 303, G686–G695. doi: 10.1152/ajpgi.00140.2012, PMID: 22790597

[ref78] Singh B. Ryan H. Kredo T. Chaplin M. Fletcher T. (2021). Chloroquine or hydroxychloroquine for prevention and treatment of COVID-19. Cochrane Database Syst. Rev. 2021:CD013587. doi: 10.1002/14651858.CD013587.pub2, PMID: 33624299 PMC8094389

[ref79] Spinner C. D. Gottlieb R. L. Criner G. J. Arribas López J. R. Cattelan A. M. Soriano Viladomiu A.. (2020). Effect of remdesivir vs standard care on clinical status at 11 days in patients with moderate COVID-19: a randomized clinical trial. JAMA 324, 1048–1057. doi: 10.1001/jama.2020.16349, PMID: 32821939 PMC7442954

[ref80] Stenstad H. Ericsson A. Johansson-Lindbom B. Svensson M. Marsal J. Mack M.. (2006). Gut-associated lymphoid tissue-primed CD4+ T cells display CCR9-dependent and -independent homing to the small intestine. Blood 107, 3447–3454. doi: 10.1182/blood-2005-07-2860, PMID: 16391017

[ref81] Taherifard E. Mortazavi R. Mokhtari M. Taherifard A. Kiani Salmi S. Taherifard E. (2022). Cytomegalovirus gastritis in a patient with severe acute respiratory syndrome coronavirus 2 infection: a case report and literature review. Respir. Med. Case. Rep. 37:101644. doi: 10.1016/j.rmcr.2022.101644, PMID: 35392550 PMC8975752

[ref82] Taxonera C. Fisac J. Alba C. (2021). Can COVID-19 trigger De novo inflammatory bowel disease? Gastroenterology 160, 1029–1030. doi: 10.1053/j.gastro.2020.11.026, PMID: 33221408 PMC7676850

[ref83] v’kovski P. Kratzel A. Steiner S. Stalder H. Thiel V. (2021). Coronavirus biology and replication: implications for SARS-CoV-2. Nat. Rev. Microbiol. 19, 155–170. doi: 10.1038/s41579-020-00468-6 33116300 PMC7592455

[ref84] Van Doorn A. S. Meijer B. Frampton C. M. A. Barclay M. L. de Boer N. K. H. (2020). Systematic review with meta-analysis: SARS-CoV-2 stool testing and the potential for faecal-oral transmission. Aliment. Pharm. Ther. 52, 1276–1288. doi: 10.1111/apt.16036, PMID: 32852082 PMC7461227

[ref85] Vanella G. Capurso G. Burti C. Fanti L. Ricciardiello L. Souza Lino A.. (2021). Gastrointestinal mucosal damage in patients with COVID-19 undergoing endoscopy: an international multicentre study. BMJ Open Gastroenterol. 8:e000578. doi: 10.1136/bmjgast-2020-000578, PMID: 33627313 PMC7907837

[ref86] Wang H. Dai W. Feng X. Zhou Q. Wang H. Yang Y.. (2018). Microbiota composition in upper respiratory tracts of healthy children in Shenzhen, China, differed with respiratory sites and ages. Biomed. Res. Int. 2018, 6515670–6515678. doi: 10.1155/2018/6515670, PMID: 30013985 PMC6022278

[ref87] Wang J. Li F. Wei H. Lian Z. X. Sun R. Tian Z. (2014). Respiratory influenza virus infection induces intestinal immune injury via microbiota-mediated Th17 cell-dependent inflammation. J. Exp. Med. 211, 2397–2410. doi: 10.1084/jem.20140625, PMID: 25366965 PMC4235643

[ref88] Wang F. Wang H. Fan J. Zhang Y. Wang H. Zhao Q. (2020). Pancreatic injury patterns in patients with coronavirus disease 19 pneumonia. Gastroenterology 159, 367–370. doi: 10.1053/j.gastro.2020.03.055, PMID: 32247022 PMC7118654

[ref89] Wang Y. Zhang D. Du G. Du R. Zhao J. Jin Y.. (2020). Remdesivir in adults with severe COVID-19: a randomised, double-blind, placebo-controlled, multicentre trial. Lancet 395, 1569–1578. doi: 10.1016/S0140-6736(20)31022-9, PMID: 32423584 PMC7190303

[ref90] Wei P. F. (2020). Diagnosis and treatment protocol for novel coronavirus pneumonia (trial version 7). Chin. Med. J. 133, 1087–1095. doi: 10.1097/CM9.0000000000000819, PMID: 32358325 PMC7213636

[ref91] Wrapp D. Wang N. Corbett K. S. Goldsmith J. A. Hsieh C. L. Abiona O.. (2020). Cryo-EM structure of the 2019-nCoV spike in the prefusion conformation. bioRxiv 367, 1260–1263. doi: 10.1101/2020.02.11.944462, PMID: 32075877 PMC7164637

[ref92] Wruck W. Adjaye J. (2020). SARS-CoV-2 receptor ACE2 is co-expressed with genes related to transmembrane serine proteases, viral entry, immunity and cellular stress. Sci. Rep. 10:21415. doi: 10.1038/s41598-020-78402-2, PMID: 33293627 PMC7723043

[ref93] Xiao F. Tang M. Zheng X. Liu Y. Li X. Shan H. (2020). Evidence for gastrointestinal infection of SARS-CoV-2. Gastroenterology 158, 1831–1833.e3. doi: 10.1053/j.gastro.2020.02.055, PMID: 32142773 PMC7130181

[ref94] Xie X. P. Sheng L. P. Han C. Q. Jin Y. Bai T. Lin R.. (2022). Features of capsule endoscopy in COVID-19 patients with a six-month follow-up: a prospective observational study. J. Med. Virol. 94, 246–252. doi: 10.1002/jmv.27308 34460118 PMC8662114

[ref95] Yan R. Zhang Y. Li Y. Xia L. Guo Y. Zhou Q. (2020). Structural basis for the recognition of SARS-CoV-2 by full-length human ACE2. Science 367, 1444–1448. doi: 10.1126/science.abb2762, PMID: 32132184 PMC7164635

[ref96] Ye X. T. Luo Y. L. Xia S. C. Sun Q. F. Ding J. G. Zhou Y.. (2020). Clinical efficacy of lopinavir/ritonavir in the treatment of Coronavirus disease 2019. Eur. Rev. Med. Pharmacol. Sci. 24, 3390–3396. doi: 10.26355/eurrev_202003_20706 32271456

[ref97] Ye Q. Wang B. Zhang T. Xu J. Shang S. (2020). The mechanism and treatment of gastrointestinal symptoms in patients with COVID-19. Am. J. Physiol. Gastrointest. Liver Physiol. 319, G245–G252. doi: 10.1152/ajpgi.00148.2020, PMID: 32639848 PMC7414235

[ref98] Yeoh Y. K. Zuo T. Lui G. C. Zhang F. Liu Q. Li A. Y.. (2021). Gut microbiota composition reflects disease severity and dysfunctional immune responses in patients with COVID-19. Gut 70, 698–706. doi: 10.1136/gutjnl-2020-323020, PMID: 33431578 PMC7804842

[ref99] Yoshida N. Hirose R. Watanabe M. Yamazaki M. Hashimoto S. Matsubara S.. (2020). A case of urgent colonoscopic hemostasis of a cecal hemorrhagic ulceration in a patient receiving heparin for COVID-19 coagulopathy. JGH Open 5, 160–162. doi: 10.1002/jgh3.12435, PMID: 33490630 PMC7812454

[ref100] Zamboni P. Bortolotti D. Occhionorelli S. Traina L. Neri L. M. Rizzo R.. (2022). Bowel ischemia as onset of COVID-19 in otherwise asymptomatic patients with persistently negative swab. J. Intern. Med. 291, 224–231. doi: 10.1111/joim.13385, PMID: 34437741 PMC8662187

[ref101] Zang R. Gomez Castro M. F. McCune B. T. Zeng Q. Rothlauf P. W. Sonnek N. M.. (2020). TMPRSS2 and TMPRSS4 promote SARS-CoV-2 infection of human small intestinal enterocytes. Sci. Immunol. 5:eabc3582. doi: 10.1126/sciimmunol.abc3582 32404436 PMC7285829

[ref102] Zeng W. Qi K. Ye M. Zheng L. Liu X. Hu S.. (2022). Gastrointestinal symptoms are associated with severity of coronavirus disease 2019: a systematic review and meta-analysis. Eur. J. Gastroenterol. Hepatol. 34, 168–176. doi: 10.1097/MEG.0000000000002072, PMID: 33470700

[ref103] Zhang L. J. Fan H. Chen R. Zhu X. W. Wang W. Z. Cui D. D.. (2020). To discuss the rational application of Qingfeipaidu decoction from clinical practice. J. Tradit. Chin. Med. 61, 1573–1577. doi: 10.13288/j.11-2166/r.2020.18.003

[ref104] Zhang H. Shao B. Dang Q. Chen Z. Zhou Q. Luo H.. (2021). Pathogenesis and mechanism of gastrointestinal infection with COVID-19. Front. Immunol. 12:674074. doi: 10.3389/fimmu.2021.674074, PMID: 34858386 PMC8631495

[ref105] Zhang Y. Xiao M. Zhang S. Xia P. Cao W. Jiang W.. (2020). Coagulopathy and antiphospholipid antibodies in patients with COVID-19. N. Engl. J. Med. 382:e38. doi: 10.1056/NEJMc2007575, PMID: 32268022 PMC7161262

[ref106] Zou J. Li L. Zheng P. Liang W. Hu S. Zhou S.. (2022). Ultrapotent neutralizing antibodies against SARS-CoV-2 with a high degree of mutation resistance. J. Clin. Invest. 132:e154987. doi: 10.1172/JCI154987, PMID: 35108220 PMC8843702

[ref107] Zuo T. Wu X. Wen W. Lan P. (2021). Gut microbiome alterations in COVID-19. Genom. Proteom. Bioinformat. 19, 679–688. doi: 10.1016/j.gpb.2021.09.004, PMID: 34560321 PMC8478109

